# A prospective observational study of head-of-bed adjustment for patients following elective craniotomy based on cerebral autoregulation

**DOI:** 10.3389/fmed.2025.1713881

**Published:** 2026-01-12

**Authors:** Yan Li, Yuyao Huang, Meihua Mei, Yufang Wang, Jingchao Li, Mingli Yao, Bin Ouyang, Lei Shi, Lingyan Wang

**Affiliations:** 1 Neurosurgical Intensive Care Unit (NSICU), The First Affiliated Hospital of Sun Yat-sen University, Guangzhou, China; 2 Intensive Care Unit (ICU), Dongguan People’s Hospital, Dongguan, China

**Keywords:** cerebral autoregulation, elective craniotomy, head-of-bed, ICP, multimode monitoring

## Abstract

**Background:**

While head-of-bed (HOB) elevation is standard in neurocritical care for managing intracranial hypertension, its optimal angle for patients after elective craniotomy remains undefined. This study aimed to evaluate the effects of different HOB angles on cerebral hemodynamics, oxygenation, and cerebral autoregulation (CA) in patients during the early postoperative period following elective craniotomy.

**Methods:**

In a prospective observational study, 21 patients underwent sequential positioning at HOB 45°, 30°, 15°, and 0°. Each angle was maintained for 15 min, with multimodal data including intracranial pressure (ICP), cerebral perfusion pressure (CPP), mean flow velocity (mFV) and pulsatility index (PI) of the middle cerebral artery (MCA) M1 segment, tissue oxygenation index (TOI) and the mean flow index (Mx-a) for CA recorded during the final 5 min. Linear mixed-effects models assessed the main effects of HOB angle, baseline ICP status (Elevated: >15 mmHg vs. Normal), and baseline CA status (Impaired: Mx-a > 0.3 vs. Intact).

**Results:**

The 0° position significantly increased ICP (coefficient +8.95 mm Hg, 95% CI 7.02 to 10.88 *p* < 0.001) and decreased CPP (coefficient −7.65 mm Hg, 95% CI −13.13 to −2.17 *p* < 0.05). Conversely, 45° elevation significantly lowered ICP (coefficient−1.64 mm Hg, 95% CI −2.87 to −0.41). A significant reduction in Mx-a was observed at 0° (coefficient −0.13, 95% CI −0.25 to −0.007, *p* < 0.05). Both the 15° and 0° postures were associated with significant decreases in PI (coefficient −0.12, 95% CI −0.21 to −0.03, *p* < 0.05 and coefficient −0.13, 95% CI −0.23 to −0.03, *p* < 0.05, respectively). TOI remained unchanged across all positions. The Elevated ICP group maintained higher ICP and lower CPP throughout. Baseline impairment of CA (Mx-a > 0.3) was independently and significantly associated with an increase in the Mx-a index itself during the study period (coefficient +0.26, 95% CI 0.13 to 0.40, *p* < 0.001).

**Conclusion:**

HOB significantly modulates cerebral hemodynamics after elective craniotomy. The 0° position is particularly detrimental, elevating ICP, reducing CPP, and potentially exhausting cerebrovascular regulatory reserve. Patient’s baseline ICP and CA status are key determinants of their hemodynamic response. Postoperative management must extend beyond the prevention of intracranial hypertension to also include the vigilant avoidance of CA impairment.

## 1 Introduction

The management of head-of-bed (HOB) elevation is a cornerstone of neurocritical care, primarily guided by evidence from traumatic brain injury (TBI) and other conditions characterized by intracranial hypertension. In these settings, a 30° HOB elevation is widely adopted to facilitate venous drainage, lower intracranial pressure (ICP), and optimize cerebral perfusion pressure (CPP) (1, 2). However, the direct extrapolation of this practice to patients recovering from elective craniotomy is questionable. Unlike classic TBI patients, this specific population may not present with significant intracranial hypertension in the immediate postoperative period, often due to intraoperative cerebrospinal fluid loss and the decompressive effect of the surgery itself. Consequently, the routine application of a standardized 30° HOB position for this distinct group lacks robust evidence and may not represent the optimal strategy.

The determination of an optimal HOB angle is critical, as inappropriate positioning can increase the risk of secondary brain injury (3, 4). This risk persists even when conventional metrics like ICP and CPP appear normal, particularly in the context of impaired cerebral autoregulation (CA) (5). In patients with brain tumors or cerebrovascular diseases, the underlying pathology frequently disrupts CA, and surgical intervention can further exacerbate this dysregulation. The postoperative period is thus characterized by dynamic shifts in cerebral compliance and hemodynamics. Under these conditions, a fixed HOB elevation that does not account for individual patient physiology could inadvertently induce cerebral hemodynamic instability or other effects, potentially compromising outcomes.

## 2 Manuscript formatting

### 2.1 Patient selection

A prospective observational study was conducted between January 2022 and December 2024. We enrolled post-pubertal patients (≥12 years) undergoing elective craniotomy who required external ventricular drainage (EVD) for obstructive hydrocephalus and intracranial pressure monitoring. Exclusion criteria comprised: hemodynamic instability; inadequate temporal acoustic window for transcranial Doppler (TCD) monitoring; conditions interfering with reliable near-infrared spectroscopy (NIRS) signal acquisition (including frontal scalp swelling, subcutaneous hematoma, subdural/epidural hematoma, subdural effusion, or frontal intracranial gas accumulation); and patients who had undergone decompressive craniectomy.

The study protocol was approved by the Ethics Committee of Clinical Research and Experimental Animals of the First Affiliated Hospital of Sun Yat-sen University (Ethical Approval No. [2022] 639). Prior to enrollment and the scheduled surgery, written informed consent was obtained from all participants or their legally authorized representatives. For adolescent participants (12–17 years), written informed consent was obtained from both parents or legally authorized guardians, and additional written assent was obtained from the adolescent participants themselves.

### 2.2 Physiological monitoring

All patients received standardized management in the neurosurgical intensive care unit. Continuous hemodynamic monitoring included electrocardiography, pulse oximetry, and invasive arterial pressure measurement via the radial artery. The arterial pressure transducer was consistently positioned at the level of the mid-axillary line and maintained at this position throughout postural changes to ensure accurate CPP calculation.

Normovolemia and cardiac function were assessed at bedside using ultrasonography. Assessment criteria included: left ventricular ejection fraction (LVEF) > 55%; inferior vena cava (IVC) diameter between 1 and 2 cm, measured in the subxiphoid view approximately 1–2 cm caudal to the hepatic vein-IVC junction; and IVC collapsibility index (IVC-CI) below 50% for spontaneously breathing patients or below 18% for mechanically ventilated patients. Central venous pressure (CVP) was maintained ≥5 cm H₂O. For mechanically ventilated patients, ventilation parameters were set at tidal volume 8 mL/kg (based on ideal body weight) with positive end-expiratory pressure (PEEP) of 5 cm H₂O. All patients were maintained within strict physiological ranges: peripheral oxygen saturation >98%, PO₂90–150 mm Hg, normocapnia (PaCO₂ 35–45 mm Hg), normothermia (36.0–37.2 °C maintained through physical cooling), serum glucose 8–10 mmol/L, and sodium 135–145 mmol/L. If these predefined targets were not met upon initial assessment, a standardized protocol was initiated to achieve physiological stabilization prior to study enrollment. This included adjusting FiO₂, and ventilator parameters (PEEP, respiratory rate, and tidal volume) to correct deviations in PaO₂ or PaCO₂, followed by a confirmatory arterial blood gas analysis after 30 min of stable settings. Physical cooling or warming pad was initiated if needed to achieve normothermia.

### 2.3 Cerebral monitoring parameters

ICP was continuously monitored through the EVD system (Medtronic Inc., Minneapolis, MN, USA) with the pressure transducer zeroed at the level of the external auditory meatus, The EVD was clamped during data acquisition periods to ensure accurate ICP measurement. Cerebral blood flow velocity was measured bilaterally using TCD (EMS-9D PRO, Delica Medical Equipment Co, Ltd., Shenzhen, China) with a 2 MHz pulsed-wave probe secured by a fixation headframe over the temporal window, insonating the M1 segment of the middle cerebral artery at 50–55 mm depth, Cerebral blood flow velocity was continuously monitored via TCD, The time-averaged mean flow velocity (mFV) was automatically calculated by the TCD device over each consecutive cardiac cycle. Regional cerebral oxygenation was assessed using NIRS (EGOS-600A, Aiqin Biomedical Electronics Co, Ltd., Suzhou, China) with bilateral frontal probes monitoring tissue oxygenation index (TOI) and tissue hemoglobin index (THI).

### 2.4 Study protocal and data analysis

**Figure 1:**
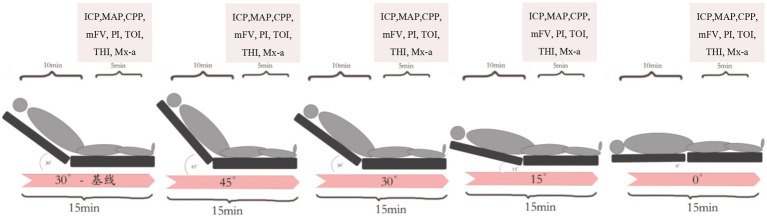
The study protocol and timing of data collection. ICP, intracranial pressure; MAP, mean arterial pressure; CPP, cerebral perfusion pressure; mFV, mean cerebral blood flow velocity; PI, pulsatility index; TOI, tissue oxygenation index; THI, tissue hemoglobin concentration index; Mx-a, mean arterial flow index.

The study protocol was initiated after all the aforementioned physiological parameters were consistently maintained within the target ranges. Beginning at 30° HOB elevation with the head in neutral position, patients were sequentially positioned to 45°, 30°15°, and 0°HOB angles. Each position was maintained for 15 min to ensure physiological stabilization, with multimodal data recorded during the final 5 min of each epoch, the study protocol and timing of data collection are shown in Figure 1. Interventions that could confound results, such as suctioning, repositioning, feeding, and osmotic therapy, were temporarily suspended. Sedative, vasoactive, and antihypertensive medications, as well as ventilator settings, remained unchanged throughout the study. The experiment was paused if MAP decreased by >15% from baseline, ICP exceeded 30 mmHg, or TOI decreased by >15% from baseline.

Data from the surgical hemisphere were utilized for all primary analyses of both TCD and NIRS parameters. Continuous monitoring data acquired during the final 5 min at each HOB angle, were digitized and processed using the ICM + software platform (Cambridge Enterprise Ltd., UK). The assessment of dynamic cerebral autoregulation was performed using the mean flow index (Mx-a). The methodology for calculating Mx-a was as follows, the raw analog signals for MAP and mFV were first processed with a 10-s moving average filter. The Mx-a index was then computed as the moving Pearson correlation coefficient between these processed MAP and mFV signals. The coefficient was updated every 10 s, based on a 5-min (300-s) moving window, which provided 30 consecutive data points for each correlation calculation. This protocol ensures both adequate physiological stabilization and the acquisition of a sufficient time series for robust dynamic autoregulation analysis (5).

Based on established literature, cerebral autoregulation was classified as impaired if the mean Mx-a value over the analysis period was > 0.3, and intact if it was ≤ 0.3 (6, 7). Patients were stratified *post-hoc* into two groups based on their baseline measurements at the 30° head-of-bed position. The ‘Elevated ICP’ group was defined by an ICP > 15 mm Hg, while the ‘Impaired Cerebral Autoregulation (CA)’ group was defined by an Mx-a index > 0.3.

### 2.5 Statistical analysis

The sample size was not predetermined by a formal power calculation. As an exploratory physiological study focusing on detailed within-subject responses to posture change, we included all eligible consecutive patients who completed the standardized protocol with high-quality multimodality monitoring data during the study period. Data were analyzed using SPSS (Version 25.0, IBM, Armonk, NY). Continuous variables are presented as mean ± standard deviation for normally distributed data or median with interquartile range for non-normally distributed data, with normality assessed using the Shapiro–Wilk test. To analyze the repeated measures across HOB angles, a linear mixed-effects model was employed. The model included patients as random effects (random intercept), with HOB angle (0°, 15°, 30°, 45°), baseline ICP status (normal vs. elevated), and baseline CA status (impaired vs. intact) as fixed effects. Statistically significant and clinically relevant interaction terms between these fixed effects were considered for inclusion. Model fit was compared using the Bayesian Information Criterion (BIC). For each final model, the estimated coefficient (*β*) for significant predictors is reported alongside its 95% confidence interval (CI) and *p*-value. A *p*-value of < 0.05 was considered statistically significant.

## 3 Results

### 3.1 Characteristics of the study population

**Figure 2:**
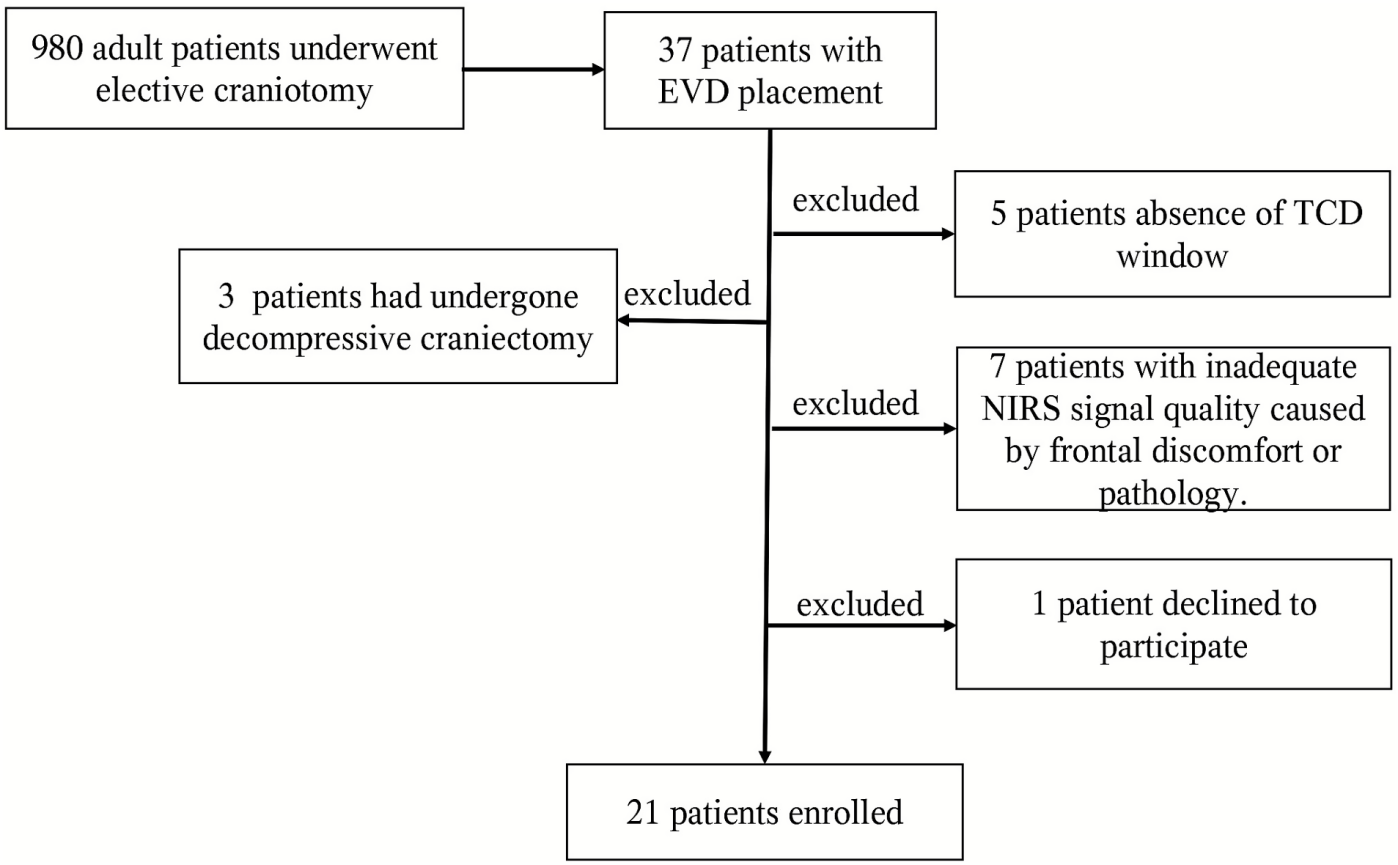
Flow diagram of study participants. EVD, external ventricular drainage; TCD, transcranial Doppler; NIRS, near-infrared spectroscopy.

Finally, the study included a total of 21 patients. The patient enrollment flowchart is presented in Figure 2. Table 1 provides the demographics of each patient. The lesions primarily affected the diencephalon, pontocerebellar angle region, and ventricular system. The findings from postoperative head CT scans primarily included cerebral edema, intracranial hemorrhage, subarachnoid hemorrhage, and pneumocephalus. At a baseline HOB elevation of 30°, ICP was above 15 mm Hg in 7 patients and below 15 mm Hg in the other 14 patients. Mx-a above 0.3 was observed in 8 patients, while 13 patients exhibited intact CA (Mx-a ≤ 0.3). Physiological parameters, including body temperature, PaO₂, PaCO₂, blood glucose, and serum sodium levels, et al., at the time of the protocol implementation were showed in Table 2. The absolute values of key monitoring parameters across the four tested HOB elevations are summarized in Table 3. There was only one patient experienced a transient ICP elevation above 30 mm Hg upon lowering the HOB to 0 degrees. No other adverse events occurred.

**Table 1:** Patients’ demographics and postoperative CT findings.

Case no.	Age (yrs)	Sex	GCS	Diagnosis	Lesion site	CT fingdings
1	41	F	14	Astrocytoma, IDH-mutant, CNS WHO Grade 2	Frontal Lobe	CE
2	31	M	10	Glioblastoma, IDH-wildtype, CNS WHO Grade 4	Diencephalon	CE, IPH
3	55	M	11	Vestibular Schwannoma	PCA Region	CE, Hydrocephalus, SAH
4	47	F	3 T	Anaplastic Meningioma, CNS WHO Grade 3	Cerebellum	CE, CVST
5	51	F	7 T	Pilocytic Astrocytoma, CNS WHO Grade 1	Diencephalon	CE, IPH, BH
6	24	M	7 T	Astrocytoma, IDH-mutant, CNS WHO Grade 4	Corpus Callosum, VS	CE, IPH, IVH
7	60	M	15	Trigeminal Schwannoma	PCA Region	Hydrocephalus, EH
8	54	M	8	Astrocytoma, IDH-mutant, CNS WHO Grade 2	Diencephalon	CE, IPH, Hydrocephalus
9	71	M	10	Oligodendroglioma, IDH-mutant and 1p/19q-codeleted, CNS WHO Grade 2	Parietal Lobe, VS	CE, Hydrocephalus, IPH, IVH
10	12	M	5 T	Dysembryoplastic neuroepithelial tumor (DNET) (CNS WHO Grade 1)	CE, Hydrocephalus, TH	CE, Hydrocephalus, TH
11	35	M	15	Papillary Craniopharyngioma CNS WHO Grade 1	Sellar Region, VS	Hydrocephalus
12	41	M	15	Atypical Meningioma, CNS WHO Grade 2	Parietal Lobe, VS	CE
13	34	M	11	Astrocytoma, IDH-mutant, CNS WHO Grade 4	VS	CE, SAH
14	51	F	10	Atypical Meningioma, CNS WHO Grade 2	Sellar Region	SAH, ICH
15	50	F	6 T	Meningioma, CNS WHO Grade 1	PCA Region	CE, SAH
16	29	M	15	Glioblastoma, IDH-wildtype, CNS WHO Grade 4	VS	CE
17	44	F	14	Atypical Meningioma, CNS WHO Grade 2	Sellar Region	CE, Hydrocephalus, SAH, ICH, CI
18	55	F	14	Meningioma, CNS WHO Grade 1	Parasellar Region	CE
19	62	M	9	Astrocytoma, IDH-mutant, CNS WHO Grade 2	Diencephalon	CE, SAH, IPH, CI
20	44	M	9	Vestibular schwannoma	PCA Region	Hydrocephalus, SAH, ICH
21	15	M	14	Ganglioglioma, CNS WHO Grade 1	Cerebellum	TH, BH

BH, brain herniation; CE, cerebral edema; CI, cerebral infarction; CT, computed tomography; CVST, cerebral venous sinus thrombosis; EH, epidural hematoma; GCS, Glasgow Coma Scale; ICH, intracerebral hemorrhage; IPH, intraparenchymal hemorrhage; IVH, intraventricular hemorrhage; PCA, pontocerebellar angle; SAH, subarachnoid hemorrhage; TH, tumor hemorrhage; VS, ventricular system.

**Table 2:** Baseline status and physical parameters at protocal implementation.

Baseline data	
Age, yr	43.32 ± 14.88
Male sex, *n* (%)	14 (66.6)
Machine ventilation, *n* (%)	7 (33.3)
The time interval from ICU admission to protocol initiation, *n* (%)	
Between 0–1 h	3 (14.3)
Between 1–3 h	10 (47.6)
Between 3–5 h	8 (38.1)
Baseline ICP, *n* (%)	
<15 mmHg	14 (66.7)
15-20 mmHg	5 (23.8)
>20 mmHg	2 (9.5)
Baseline Mx-a, *n* (%)	
≤0.3	13 (61.9)
>0.3	8 (38.1)
Physical parameters	
Body temperature (°C)	37.1 [36.6–37.9]
HR (rate/min)	81 [74–93]
SBP (mm Hg)	140 [124.7–147.2]
DBP (mm Hg)	62.5 [59–70.3]
MAP (mm Hg)	80.1 [66.6–87.9]
PaO₂ (mm Hg)	140 [109–165]
PaCO₂ (mm Hg)	37.5 [31.7–43.2]
SpO₂	100 [99–100]
O/I	363.5 [314.7–491.8]
CVP (cm H₂O)	7 [5–7]
EF (%)	69.5 [65–76]
IVCmax (cm)	1.6 [1.5–1.9]
IVC-CI (%)	0.22 [0.16–0.36]
Hb (g/L)	92 [85–113]
Blood glucose (mmol/L)	8.5 [7.2–9.9]
Serum sodium (mmol/L)	139.5 [138–144.5]
Arterial lactate (mmol/L)	1.55 [1.07–2.6]

ICP, intracranial pressure; Mx-a, mean flow index; HR, heart rate; SBP,systolic blood pressure; DBP,diastolic blood pressure; MAP, mean arterial pressure; PaO2, partial pressure of arterial oxygen; PaCO2, partial pressure of arterial carbon dioxide; SpO2,peripheral oxygen saturation; O/I,oxygenation index; CVP, central venous pressure; EF, ejection fraction; IVCmax, maximum inferior vena cava diameter; IVC-CI, inferior venacava collapsibility index; Hb, hemoglobin.

**Table 3:** Monitoring parameters at different HOB positions.

Physical parameters (*n* = 21)	45°	30°	15°	0°
ICP (mm Hg)	10.82 ± 7.31	12.20 ± 6.83	14.85 ± 6.43	19.72 ± 5.95
MAP (mm Hg)	86.92 ± 11.30	86.74 ± 13.23	87.24 ± 12.52	87.56 ± 13.83
CPP (mm Hg)	76.02 ± 15.73	74.46 ± 15.84	72.46 ± 14.91	67.72 ± 14.16
TOI	61.91 ± 6.24	61.96 ± 5.99	61.95 ± 6.84	62.04 ± 5.17
THI	0.92 ± 0.53	0.92 ± 0.53	0.95 ± 0.49	0.98 ± 0.50
mFV (cm/s)	60.86 ± 22.92	62.99 ± 23.83	62.67 ± 25.15	63.28 ± 23.04
PI	1.38 ± 0.74	1.29 ± 0.59	1.21 ± 0.41	1.21 ± 0.43
Mx-a	0.16 ± 0.32	0.21 ± 0.22	0.12 ± 0.24	0.10 ± 0.31

Data are presented as mean ± standard deviation.

ICP, intracranial pressure; MAP, mean arterial pressure; CPP, cerebral perfusion pressure; TOI, tissue oxygenation index; THI, tissue hemoglobin index; mFV, mean flow velocity; PI, pulsatility index; Mx-a, mean flow index.

### 3.2 Effect of HOB position change on ICP, CPP, THI, TOI, mFV, PI and Mx-a

According to the linear mixed models, HOB posture had significant effects on ICP, CPP, THI, TOI, PI and Mx-a (Table 4). Alterations in head posture from the basal angle of 30° exerted a significant influence on ICP and CPP. Positioning the head at 0° (flat supine) was associated with a substantial increase in ICP (coefficient +8.95 mm Hg, 95% CI 7.02 to 10.88, *p* < 0.001) and a concurrent decrease in CPP (coefficient −7.65 mm Hg, 95% CI −13.13 to −2.17, *p* < 0.05). Conversely, the 45° posture was associated with a significant reduction in ICP (coefficient −1.64 mm Hg, 95% CI −2.87 to −0.41, *p* < 0.05). A 15° head elevation also significantly increased ICP compared to the 30° reference (coefficient +3.04 mm Hg, 95% CI 1.78 to 4.30, *p* < 0.001).

**Table 4:** Linear random mixed effect model for ICP, CPP, THI, TOI, mFV, PI and Mx-a.

Fixed effects parameters		Dependent variables						
		ICP	CPP	TOI	THI	mFV	PI	Mx-a
Head posture (Reference: 30°)	45°	−1.64 (−2.87 to −0.41)*	+0.85 (−2.48 to 4.20)	−0.02 (−0.51 to 0.45)	−0.003 (−0.24 to 0.17)	−1.49 (−4.92 to 1.93)	+0.11 (0.02 to 0.19)*	−0.08 (−0.20 to 0.03)
	15°	+3.04 (1.78 to 4.30) **	−1.97 (−6.57 to 2.61)	−0.002 (−0.62 to 0.61)	+0.02 (−0.01 to 0.07)	+0.91 (−2.67 to 4.50)	−0.12 (−0.21 to −0.03) *	−0.09 (−0.22 to 0.04)
	0°	+8.95 (7.02 to 10.88) **	−7.65 (−13.13 to −2.17) *	+0.05 (−0.64 to 0.76)	+0.05 (0.006 to 0.10)*	+1.75 (−1.86 to 5.36)	−0.13 (−0.23 to −0.33) *	−0.13 (−0.25 to −0.007) *
ICP > 15 mmHg on baseline (the Elevated ICP group)		+11.58 (7.11 to 16.06)**	−14.34 (−27.21 to −1.46) *	+0.66 (−3.27 to 4.60)	+0.10 (−0.30 to 0.51)	+8.13 (−8.05to 24.33)	−0.23 (−0.62 to 0.15)	−0.14 (−0.34 to 0.05)
Mx-a > 0.3 on baseline (the Impaired CA group)		−0.58 (−8.06 to 6.89)	−6.0 (−21.47 to 9.45)	+2.76 (−1.31 to 6.84)	−0.03 (−0.49 to 0.42)	+4.94 (−12.04 to 21.93)	+0.25 (−0.15 to 0.66)	+0.26 (0.13 to 0.40)**

ICP, intracranial pressure; CPP, cerebral perfusion pressure; mFV, mean flow velocity; PI, pulsatility index; TOI, tissue oxygenation index; THI, tissue hemoglobin concentration index; Mx-a, mean arterial flow index.

All models included fixed effects for head posture (0°, 15°, 45° vs. 30° reference), baseline ICP status (Elevated ICP group, >15 mmHg vs. Normal ICP group), and baseline CA status (Impaired CA group, Mx-a > 0.3 vs. Intact CA group). Data are presented as estimated coefficient (95% Confidence Interval).

*p < 0.05, **p < 0.001 vs. reference covariate at baseline.

Furthermore, head posture significantly affected the PI and Mx-a. Both the 15° and 0° postures were associated with small but significant decreases in PI (coefficient −0.12, 95% CI −0.21 to −0.03, *p* < 0.05 and coefficient −0.13, 95% CI −0.23 to −0.03, *p* < 0.05, respectively). A significant reduction in Mx-a was observed at the 0° posture (coefficient −0.13, 95% CI −0.25 to −0.007, *p* < 0.05), the changes in Mx-a at 15° and 45° postures were not statistically significant compared to 30°. And, no statistically significant effects of head posture were observed on TOI, THI, or mFV.

### 3.3 Effects of baseline ICP and CA status

Patients in the Elevated ICP group (ICP > 15 mm Hg at baseline) demonstrated markedly different hemodynamics compared to the Normal ICP group. As expected, the Elevated ICP group was associated with a significantly higher model-estimated ICP (coefficient +11.58 mm Hg, 95% CI 7.11 to 16.06, *p* < 0.001) and a significantly lower CPP (coefficient −14.34 mm Hg, 95% CI −27.21 to −1.46, *p* < 0.05). Regarding baseline status, the model estimated that the Elevated ICP group had an average Mx-a value 0.14 points lower than the Normal ICP group across all postures; however, this difference was not statistically significant (95% CI −0.34 to 0.05). No other parameters, including TOI, THI, FV, PI or Mx-a, showed statistically significant associations with baseline ICP status.

The model indicated that baseline impairment of cerebral autoregulation (Mx-a > 0.3) was independently and significantly associated with an increase in the Mx-a index itself during the study period (coefficient +0.26, 95% CI 0.13 to 0.40, *p* < 0.001). However, no other outcome variables, including ICP, CPP, TOI, THI, mFV or PI, exhibited statistically significant associations with the baseline CA status.

## 4 Discussion

This study employed linear random mixed-effects models to dissect the independent influences of head posture, baseline ICP, and CA status on a comprehensive set of cerebral hemodynamic and oxygenation parameters. Our findings not only reinforce established principles of neurocritical care but also unveil nuanced insights, particularly relevant to the early postoperative period following elective craniotomy.

### 4.1 Head posture: a potent and parameter-specific modulator

The most robust and clinically significant findings pertain to the effect of HOB angle. As consistently demonstrated in the literature and confirmed here, the flat supine position (0°) presents the most deleterious hemodynamic profile, characterized by a significant increase in ICP and a concomitant decrease in CPP (8, 9).

Beyond these classic pressure metrics, our data reveal a more complex, posture-specific effect on cerebrovascular dynamics. The significant reduction in PI at lower angles (0° and 15°) may reflect altered distal vascular resistance or compliance under gravitational stress (10). The significant decrease in Mx-a at 0° warrants careful interpretation. While a lower Mx-a typically indicates stronger pressure reactivity (better CA), its occurrence alongside a profound ICP surge suggests an alternative pathophysiology. We posit that the marked ICP elevation at 0° may drive CPP toward the lower limit of autoregulation, exhausting vasomotor reserve and forcing cerebral arterioles into a state of maximal, passive vasodilation. The increase of PI with the decrease of HOB also confirmed this regulatory mechanism (10). In this “pressure-passive” state, the dynamic correlation between arterial pressure and flow velocity diminishes, paradoxically resulting in a lower Mx-a value. This phenomenon, where CA indices appear to “improve” at perfusion extremes, has been documented in states of critical cerebrovascular compromise and should be interpreted as a warning sign of exhausted autoregulatory capacity rather than its enhancement (11, 12). The stability of Mx-a at elevated positions (15°–45°) suggests preserved CA function within this range.

Crucially, our study found no significant effects of head posture on cerebral tissue oxygenation (TOI). This null finding stands in contrast to some previous reports that documented changes in brain tissue oxygen tension (PbtO₂) with postural changes (13). This discrepancy can likely be attributed to two key factors. First, there are fundamental methodological differences between the monitoring modalities. Our study used near-infrared spectroscopy (NIRS)-derived TOI, which provides a non-invasive, regional assessment of cortical oxygenation. In contrast, many studies reporting significant changes utilized PbtO₂ monitoring, an invasive technique that measures oxygen tension in a very localized volume of brain tissue. These two metrics reflect related but distinct physiological phenomena and are known to exhibit different sensitivities to various insults (14, 15). Furthermore, the inherent limitations of NIRS for monitoring cerebral oxygenation should be considered when interpreting the TOI data. A primary limitation is its susceptibility to signal contamination from extracranial blood flow, a well-known and unavoidable constraint of the technology. Additionally, inter-individual anatomical variations in the frontal region (e.g., differences in skull thickness and frontal sinus size) can influence signal penetration and absolute TOI values, which may affect inter-subject comparisons. Second, cohort characteristics are pivotal. Our study specifically enrolled a post-elective craniotomy cohort, the majority of whom had normal or low baseline ICP and intact CA. In such a population, robust compensatory mechanisms (e.g., CA) are likely sufficient to maintain cortical oxygenation despite postural changes in CPP and ICP. In contrast, studies reporting oxygenation changes often involved cohorts with more severe initial injuries, higher rates of impaired CA, or established intracranial hypertension, where such compensatory reserves may be compromised. In these critically ill populations, physiological reserves are often overwhelmed, making cerebral oxygenation more vulnerable to hemodynamic perturbations induced by HOB elevation (16). Therefore, the absence of a significant TOI change in our study does not contradict earlier findings but rather highlights the context-specific nature of cerebral oxygenation responses.

### 4.2 The enduring influence of baseline ICP and CA

Our models confirmed that a patient’s baseline physiological state is a powerful determinant of their subsequent hemodynamic profile. Patients in the Elevated ICP group sustained significantly higher ICP and lower CPP throughout the study, underscoring the persistent nature of this pathophysiological state. Independently, patients with impaired CA at baseline (Mx-a > 0.3) demonstrated a sustained elevation in the Mx-a index, validating its role as a marker of ongoing cerebrovascular regulatory failure and aligning with its established association with worse neurological outcomes (17).

A pivotal finding of our study was the heterogeneity in baseline ICP, with the majority of patients presenting with normal or low values in the early postoperative phase after elective craniotomy. This is not a confounding factor but a deliberate reflection of the real-world pathophysiology in the early postoperative phase after elective craniotomy, often influenced by factors such as intraoperative CSF drainage. This diversity is not a confounder but a key feature that enriches the clinical interpretation of our results. It allows us to posit that the clinical implication of a posture-induced ICP change is fundamentally dependent on the patient’s starting point. For instance, an elevation in ICP from a low baseline upon moving to 0° may be beneficial by improving intracranial conditions and facilitating venous return. Conversely, the same absolute increase in a patient with pre-existing intracranial hypertension could critically compromise CPP, as our data in the Elevated ICP group suggest. This underscores the paramount importance of individualized management.

### 4.3 Clinical implications: beyond intracranial hypertension

Our results carry significant clinical implications. They robustly affirm that head elevation is a simple and effective intervention for reducing ICP. More importantly, our data reveal that HOB positioning is a critical care lever even for patients with normal or low ICP. The dramatic reductions in CPP observed at 0° in some patients highlight a previously underappreciated risk: the flat supine position can inadvertently induce impairment of CA in a vulnerable postoperative population. Therefore, management must be individualized. While higher head positions benefit patients with elevated ICP, the optimal strategy for patients with low/normal ICP is to titrate the HOB angle to a level that simultaneously ensures adequate CPP and preserves optimal CA function, thereby safeguarding the brain’s physiological reserve against hypoperfusion insults.

## 5 Limitations

This study has several limitations that must be considered when interpreting the results. First, the single-center design and relatively small sample size (*n* = 21) limit the generalizability of our findings. While this sample was sufficient to detect the large main effects reported here, it unequivocally limited the statistical power for conducting reliable subgroup analyses (e.g., intricate interactions between posture, ICP group, and CA group) or for including numerous covariates in multivariate models. Second, the fixed sequence of HOB testing, although standardized for safety and protocol consistency, may introduce carry-over effects. Third, in line with the exploratory nature of this physiological study, we did not adjust for multiple comparisons across the seven hemodynamic endpoints, which increases the risk of Type I errors; therefore, the significant findings, especially those with *p*-values near 0.05, should be interpreted as hypothesis-generating. Fourth, regarding NIRS, the technique has inherent limitations. Although, we excluded patients with major frontal pathologies, NIRS monitoring has inherent limitations, including potential contamination from extracranial blood flow and sensitivity to inter-individual anatomical variations, which we could not fully eliminate despite our strict exclusion criteria. Finally, the administration of anesthetic and vasoactive medications was guided by clinical necessity rather than a fixed research protocol. Therefore, we did not systematically control for their potential confounding effects on vascular tone across all measurements, which represents a potential source of bias.

## 6 Conclusion

In conclusion, this study provides robust, model-based evidence that head posture is a parameter-specific modulator of cerebral hemodynamics in patients following elective craniotomy. A patient’s baseline ICP and CA status are pivotal in defining their unique hemodynamic profile. Crucially, our findings underscore that postoperative management must extend beyond the prevention of intracranial hypertension to also include the vigilant avoidance of exacerbate CA function, advocating for a tailored, multimodality-monitoring-guided approach to head positioning in neurocritical care.

## Data Availability

The original contributions presented in the study are included in the article/supplementary material, further inquiries can be directed to the corresponding authors.
